# Varietal improvement options for higher rice productivity in salt affected areas using crop modelling

**DOI:** 10.1016/j.fcr.2018.08.020

**Published:** 2018-12-01

**Authors:** Ando M. Radanielson, Donald S. Gaydon, Md. Mahbubur Rahman Khan, Apurbo K. Chaki, Md. Atikur Rahman, Olivyn Angeles, Tao Li, A. Ismail

**Affiliations:** a International Rice Research Institute, Philippines; b CSIRO Agriculture and Food, Brisbane, Australia; c School of Agriculture and Food Sciences, The University of Queensland, St Lucia, Brisbane, Australia; d On-Farm Research Division, Bangladesh Agricultural Research Institute (BARI), Joydebpur, Gazipur 1701, Bangladesh

**Keywords:** Cropping systems, Genotype, Modelling, ORYZA v3, Trait selection, Water availability

## Abstract

The rice model ORYZA v3 has been recently improved to account for salt stress effect on rice crop growth and yield. This paper details subsequent studies using the improved model to explore opportunities for improving salinity tolerance in rice. The objective was to identify combinations of plant traits influencing rice responses to salinity and to quantify yield gains by improving these traits. The ORYZA v3 model was calibrated and validated with field experimental data collected between 2012 and 2014 in Satkhira, Bangladesh and Infanta, Quezon, Philippines, then used for simulations scenario considering virtual varieties possessing different combinations of crop model parameter values related to crop salinity response and the soil salinity dynamic observed at Satkhira site. Simulation results showed that (i) short duration varieties could escape end of season increase in salinity, while long duration varieties could benefit from an irrigated desalinization period occurring during the later stages of crop growth in the Satkhira situation; (ii) combining short duration growth with salt tolerance (bTR and bPN) above 12 dS m^−1^ and a resilience trait (aSalt) of 0.11 in a variety, allows maintenance of 65–70% of rice yield under increasing salinity levels of up to 16 dS m^−1^; and (iii) increasing the value of the tolerance parameter b by 1% results in 0.3–0.4% increase in yield. These results are relevant for defining directions to increase rice productivity in saline environments, based on improvements in phenology and quantifiable salt tolerance traits.

## Highlights

• Rice yield gains with improvement in salinity tolerance were quantified using field experiments and crop modelling.
• The rice variety BRRI dhan47 presented an optimum tolerance for current salinity conditions in Satkhira, Bangladesh.
• Improving salinity tolerance of IR64 by 1% resulted in a yield gain of 0.30 – 0.40% in Satkhira, Bangladesh.

## 1 Introduction

The coastal zones of Bangladesh are among the world’s most vulnerable areas to climate change. Sea level rise and reduced freshwater flow from upper catchments are the main factors leading to increasing soil and water salinity with negative effects on crop production (Dasgupta et al., 2014). More than 53% of the country’s cultivated area is exposed to salinity, with rice as the main crop (Haque, 2006; Dasgupta et al., 2009). Improving rice cropping system productivity in salt affected areas is therefore a major challenge in developing the resilience of crop production in a changing climate and maintaining the country food security.

Soil salinity dynamics are among the main biophysical factors determining timing in the rice cropping calendar for this salt-affected environment (Gaydon et al., 2014, 2018). Cropping in the wet season starts after sufficient freshwater has desalinized the upper soil layers to ensure that wet-season rice (Aman) is not affected by salinity. During the dry season, the availability of freshwater for irrigation becomes limited as the salinity of surrounding rivers water increases. The potential for producing a second rice crop in the dry ‘boro’ season currently depends on the availability of freshwater stored from the wet season. Adaptation strategies allowing rice cropping in the dry season require then setting appropriate sowing dates, together with the use of improved salt-tolerant rice varieties and freshwater management (Ismail et al., 2007; Deryng et al., 2011; Gaydon et al., 2018).

The date of sowing plays a crucial role in optimising crop production and in climate change adaptation (Deryng et al., 2011; Gaydon et al., 2014, 2018; Radanielson et al., 2015). In wheat, a loss of 57 kg ha^−1^ of yield per day has been reported with delay in sowing beyond 20th November (Krupnik et al., 2015). For wet season rice, sowing around the 30th of May has been reported to lead to maximum yield (Ahmed et al., 2014). In the dry season, Mondal et al. (2010, 2015) found that sowing around the 15th of November is optimum for ‘boro’ rice in Bangladesh. This has been supported by some recent modelling studies (Gaydon et al., 2018). The determination of the optimum cropping calendar therefore, changes with the crop, the variety and the climatic conditions. This kind of time and site-specific management is more complex when considering the complexity of salinity dynamics over time and space in the field.

Rice is one of few crops that can be successfully grown in salt-affected soils, even though it is considered sensitive to salinity. This is because growing in flooded fields allows partial desalinization of the soil and reduces the impact of salinity on crop growth and yield (Ismail et al., 2007; Singh et al., 2010). Significant efforts have been devoted to breed salt tolerant rice varieties in the recent past, and several varieties were developed through combining conventional and molecular breeding (Gregorio et al., 2002; Islam et al., 2008; Kole et al., 2015). Progress in understanding physiological mechanisms and genetic control of salinity tolerance has accelerated and enhanced the breeding of tolerant varieties (Ismail et al., 2007; Thomson et al., 2010). However with the anticipated worsening conditions of salinity caused by climate change, especially in coastal tropics, it is expected that further improvement in salinity tolerance is required in future varieties (Nicholls et al., 2016; Clarke et al., 2015; IPCC, 2014). To sustain rice production in the coastal saline areas, varieties combining tolerance to multiple stresses such as salinity, drought and submergence are required (Ismail et al., 2007, Ismail et al., 2009; Wassmann et al., 2009a; Kole et al., 2015). The development of such ‘climate ready’ varieties remains challenging and requires a multidisciplinary approach, particularly when considering the stability of these varieties across environments (Islam et al., 2015). Conventional varietal assessment using multi environment trials is limited in its ability to explore different combinations of environmental factors and crop traits. In addition, time and site-specific management of these trials are costly and time consuming. Temporal and spatial variability of salinity adds further complexity to such varietal assessment.

Simulation modelling provides a practical means for addressing this complexity. It offers an alternative to examine possible combinations of crop traits and to assess their performance in real environments, therefore accelerating selection and delivery in breeding. Process-based crop models use quantitative descriptions of various factors limiting crop productivity. By detangling crop productivity into key factors, a platform for virtual experiments is created to test hypotheses and quantify impacts of variation in environments, management and genotype on grain yield, besides other system variables like water productivity (Balwinder-Singh et al., 2015; Radanielson et al., 2015; Gaydon et al., 2018).

Crop modelling allows extrapolation of field experiment results to long-term understanding via multi-year simulation. Consideration of system performance in both historical and future conditions is possible using daily measured and generated climate data, respectively. This provides a greater insight into long-term system risk and variability than could possibly be obtained from several years of experimental results alone. Models can effectively be used to determine optimal management practices and can integrate variability in crop genotypic responses (Matthews et al., 1997; Bouman and van Laar, 2006; Bannayan et al., 2005; Li et al., 2013; Balwinder-Singh et al., 2015). Applications of models for crop improvement include evaluating the impact of specific characteristics on yield (and its season-to-season variability) and the determination of optimum ideotypes for particular production ecosystems (Sinclair and Muchow, 2001; Chapman, 2008; Chenu et al., 2008; Casadebaig et al., 2011). Characterizing target population environments is also among the most frequent uses of modelling for variety assessment (Chenu et al., 2009a). Modelling assists decision- making in breeding programs and can enhance the rate of yield gain within groups of environments (Hammer et al., 2005; Chenu et al., 2011). Studies have also demonstrated the usefulness of modelling by integrating advances in crop physiology to enhance breeding programs, particularly in improving complex traits such yield and drought tolerance (Hammer et al., 2002; Cooper et al., 2005; Chenu et al., 2009b). However, these approaches have not been sufficiently employed in rice breeding programs. The crop model ORYZA v3 (Bouman et al., 2001; Li et al., 2017) has been recently used to assess drought-tolerant traits in rainfed rice systems in South Asia (Li et al., 2013). It has also been applied to estimate climate change effects on rainfed rice systems and demonstrate the importance of modifying sowing dates to cope with climate change (Li et al., 2015).

In this study, we demonstrate the use of crop modelling to quantify the contribution of salinity tolerance and resilience traits on rice yield variability for areas with light to moderate salt stress such the Satkhira region of Bangladesh (8–12 dS m^−1^). As part of the study, we characterized the salt tolerant variety BRRI dhan47. The main objective was to define approaches and options for further improvement of this variety as well as future varieties for the region, using modelling. In salt-affected areas such as coastal Bangladesh, variability of soil salinity is driven by the interaction of climatic and environmental factors (rainfall, temperature, river salinity), with crop management (irrigation and sowing dates). Suitable varieties must exhibit specific traits to cope with these environmental and management conditions. The rice crop model ORYZA v3 (Li et al., 2017) used in this study has been recently improved to represent the effects of salinity on rice growth and yield (Radanielson et al., 2018a). We initially conducted field experiments to calibrate and validate the model’s performance in simulating genotypic variability in rice response to salinity. Scenario analyses were then performed using long-term historical climate data for Satkhira, Bangladesh, investigating the effect of early and late sowing dates with a range of virtual salinity trait combinations in a rice genotype.

## 2 Materials and methods

The initial aim of this research was to parameterise, calibrate and validate the ORYZA v3 model (Radanielson et al., 2018a; Li et al., 2017) using data from several years of field trials in different environments (Philippines and Bangladesh), with a range of existing rice varieties contrasting in their salinity tolerance. Successful model performance in this process facilitated subsequent scenario analyses, investigating performance of virtual varieties for the coastal Bangladesh environment.

### 2.1 Model calibration and validation

#### 2.1.1 Field experiments

##### 2.1.1.1 Rice genotypes

Four field experiments were conducted using three varieties of contrasting salinity tolerance: BRRI dhan47, IR64 and IR29. BRRI dhan47 is one of the salt tolerant varieties used by farmers in Bangladesh (Islam et al., 2008). The variety IR29 is generally used by breeders as a sensitive check in breeding for salinity tolerance; and IR64 is a widely known variety, frequently used as high yielding parent in breeding with intermediate salt tolerance.

##### 2.1.1.2 Field experimental design

The experiments were performed during the dry seasons of 2012–2014 and were conducted at two sites. Experiments (Expts) 1 and 2 were conducted at Infanta, Quezon Philippines (14° 45′N, 121°41′E) using the three varieties. Expts 3 and 4 were conducted at Satkhira, Bangladesh (24° 12′N’, 90°12′E) using BRRI dhan47 (Table 1). Expts 1 and 2 were established in a randomized split-plot design with three replicates. Each experiment had four treatments of irrigation management (main factor) and three varieties (sub-factors). Expts 3 and 4 had three and four irrigation management treatments, respectively, in a randomized block design with three replicates. The irrigation treatments were managed to create salt stress conditions in the field covering 4 salinity levels, corresponding to average soil salinities of 0–2 dS m^−1^, 2–4 dS m^−1^, 6–8 dS m^−1^ and higher than 10 dS m^−1^ (Table 1).

**Table 1:** Experiments used for model calibration and validation in simulating genotypic variability in responses of rice varieties to salinity. FW, irrigated with freshwater; SW, irrigated with saline water; 1 W, irrigated with alternate one week freshwater to one week saline water in Expts 1 & 2 and with one to one ratio by volume of fresh and saline water in Expts 2 and 3; 2 W, irrigated with alternate two weeks freshwater to one week saline water in Expts 1 & 2 and with mixing two volume of freshwater with one volume of saline water in Expt 4.

Experiment	Dry Season	Site	Sowing date	Transplanting date	Varieties	Soil salinity (dS m^−1^) min-max (Average)			
						FW	SW	1W	2W
Expt 1	2013	Infanta	January 26 2013	February 16, 2013	IR64, IR29, BRRI dhan47	0.8–4.6 (1.5)	1.0–14.8 (7.7)	0.5–4.0 (1.3)	0.7–7.3 (3.3)
Expt 2	2014	Infanta	January 15 2014	February 4, 2014		1–1.4 (1.1)	1.05–6.8 (3.9)	1.0–4.3 (3.2)	1.0–4.4 (3.1)
Expt 3	2013	Satkhira	December 20 2012	February 2, 2013	BRRI dhan47	0.1–6.11 (2.8)	1.0–10.4 (4.4)	1.02–4.4 (2.8)	N/A^a^
Expt 4	2014	Satkhira	January 1 2014	February 2, 2014		1.0–6.4 (3.1)	0.6–8.7 (4.0)	1.0–5.5 (2.9)	1.0–4.7 (2.9)

a N/A: data not available.

In Expts 1 and 2, four different water management treatments were used: (i) continuous irrigation with saline water (SW); (ii) continuous irrigation with freshwater (FW); (iii) weekly alternation between fresh and saline water (1 W); and two weeks fresh followed by one week saline water (2 W). Saline water was pumped from the river canal, while ground water was used as freshwater. During experimental periods, river water was slightly saline with a significant desalinization period before crop establishment. To create the desired contrasting conditions, granulated rock salt was weighed and added to the ponded water after each irrigation event for 1W, 2W and SW.

In Expts 3 and 4, treatments (i) and (ii) were the same as in Expts 1 and 2 (FW, SW). In treatments (iii) and (iv), fresh and saline water were mixed in different proportions, rather than alternating, as follows: (iii) equal volume of saline and fresh water for each irrigation (1 W), and (iv) 2:1 volume of freshwater and saline water (2 W) for each irrigation. The mixture of saline and fresh water was achieved by irrigating the field with saline water during the first half (or two thirds) of the duration and then with freshwater. Expt 3 had only three treatments; FW, SW and 1 W.

At both sites, the plots were irrigated regularly to maintain a ponded water depth of about 3 to 10 cm. In Expts 1 and 2, mean water salinity was about 0.49 and 5.5 dS m^−1^, respectively, for fresh and saline water, resulting in an increase of soil salinity from 1.5 to 7.7 dS m^-1^ (Table1). In Expts 3 and 4, the salinity of both freshwater (from a farm dam) and saline water (from the river) varied throughout the season. Salinity of the fresh water ranged from 0.65 to 1.88 dS m^−1^ with no significant variation during the crop duration. Salinity of the saline water ranged from 1.96 to 8.13 dS m^−1^, resulting in a continuous increase in soil salinity, reaching 8.4 and 10.7 dS m^−1^ by 15th and 30th of April.

##### 2.1.1.3 Management of field experiments

Fields were puddled prior to transplanting. The age of seedlings at transplanting was 21 days for Expts 1 and 2; 44 and 32 days for each of Expt 3 and Expt 4. Two to three seedlings per hill were transplanted in Expts 1 and 2 and three seedlings in Expts 3 and 4, with spacing of 20 cm x 20 cm in all experiments. The fields were managed based on local recommendations to ensure crop growth was not limited by nutrient deficiency, disease infection, or weed or pest infestation.

##### 2.1.1.4 Monitoring of field experiment and measurements

###### a) Soil and water salinity monitoring and climate data collection

Salinity of canal river water, freshwater and ponded water in the field were monitored regularly in all experiments using a calibrated hand-held EC meter (Hanna Instruments, USA). Additional systematic measurements were also performed before and after each irrigation event. In Expts 1 & 2, soil salinity was monitored continuously at 15 cm depth from the soil surface using 5TE sensors (Decagon Devices, USA). Bulk soil salinity data were recorded hourly in each plot and stored on an automatic data-logger. In Expts 3 and 4, soil salinity was monitored at the same frequency as the water salinity measurements, and was measured using a portable EC meter with one replicate per treatment. An automated Decagon weather station (model DWS, http://www.ictinternational.com/products/dws-decagon-weather-station/dws-decagon-weather-station/) was installed near the fields to record rainfall, air temperature, relative air humidity, wind speed and solar radiation on an hourly basis. Data were averaged and summed for daily values.

###### b) Crop phenology and biomass

Dates of panicle initiation, flowering and physiological maturity were recorded for each treatment in each experiment, based on the rice phenology monitor required by the ORYZA rice crop model (Li et al., 2009, 2017). Sampling for biomass was undertaken at transplanting, panicle initiation, flowering, physiological maturity and harvest time. These are key stages according to the data requirement of ORYZA v3 for model calibration and validation (Bouman et al., 2001; Li et al., 2017). Crop total aboveground biomass (WAGT) was measured from two sampling locations; each consisted of six adjacent hills covering an area of about 0.24 m^2^ in each plot. Samples were then partitioned into green leaves, dead leaves, stems, roots and panicles (when applicable) and oven dried for 48 h at 70 °C, then weighed to determine dry weights. Grain yield (GY) was determined from a 5 m^2^ area at harvest and was reported at 14% moisture content.

#### 2.1.2 Crop model calibration

##### 2.1.2.1 The model

The version of the rice crop model ORYZA v3 (Bouman et al., 2001; Li et al., 2017) including a new module to account for the effects of salinity on growth and yield was used in this study (Radanielson et al., 2018a). Parameters related to salinity responses are variety-specific as described in Radanielson et al. (2018b). The two components of salinity effect (osmotic stress and ion toxicity) are accounted for in the model. Osmotic stress is described via an equation converting soil salinity into soil osmotic potential, reducing water uptake by the crop. Responses to salt accumulation in the plant are represented via a stress factor described using a sigmoid function with two genotypic parameters defined as related to tolerance trait and resilience trait (Radanielson et al., 2018b). The function described the variation of the plant responses (photosynthesis and transpiration rate) to the soil salinity expressed as soil electrical conductivity (EC, dS m^−1^) and the presented parameters with genotypic variability. The first parameter describes the varietal tolerance to salinity, defined as the critical level of salinity at which the transpiration rate (bTR) and photosynthesis rate (bPN) decrease to 50% of their maximum, which is the inflection point of the response curve. This parameter value increases with the level of salinity tolerance of the variety. The second parameter characterizing the curve is the slope of the linear decrease of the process at the inflection point when 50% reduction is reached (aSalt). This parameter is related to the resilience of a variety, which quantifies its ability to adjust its growth to the prevailing stress. This ability allows the variety to slow down the limiting effect of salinity on its growth beyond a threshold level of salinity at which a significant decrease in growth is observed. The stress factor is then applied to both net plant photosynthesis rate and transpiration rate. An additional function was also added to the model to take into account the effect of salinity on crop phenology. This phenology stress factor has been developed empirically based on the variation in crop phenology observed in Expt 1 and 2 and has been validated with the observed phenology in Expt 3 and 4 (Supplementary Table 1). Flowering time was observed to be delayed between 3–5 days as the plant experienced salt stress during the vegetative stage (Radanielson et al., 2018b). Physiological maturity, however, was accelerated by salt stress, particularly under higher levels. The crop growth was reduced by the stress and therefore the duration of grain filling was reduced proportionally. A linear relationship was then established between the soil salinity and the duration between sowing to flowering time to capture the delay in flowering time (Eq. (1)). Another equation was used to represent the reduction in duration between flowering and physiological maturity caused by soil salinity. This then resulted in an early occurrence of physiological maturity (Eq. (1)) with soil salinity level, as indicated in the following equations:

If EC < 2 then

$$y_{fl}=1$$

$$y_{pm}=1$$

Else,

$$y_{fl}=0.15\;EC-0.09$$

(1) $$y_{pm}=-0.02\;EC+1.01$$

Where *y*_fl_ and *y*_pm_ are factors multiplied by the developmental rate of the crop, respectively, before and after flowering time; *EC* is soil electrical conductivity (dS m^−1^).

##### 2.1.2.2 Crop model parameter calibration

Data from Expt. 1 and Expt. 3 were used to calibrate the crop model parameters for the three varieties. Data from 2013 season for IR29 and IR64 were used (Expt. 1). For BRRI dhan47, calibration was performed with the 2013 data from the 2 sites (Expts. 1 & 3).

Phenological development parameters for the three varieties were computed using the phenology data from plots irrigated with freshwater (FW). The auto-calibration tool for ORYZA v3 (IRRI, 2015) was set to calibrate parameters related to biomass partitioning, leaf area expansion and drought tolerance parameters under continuous irrigation with freshwater (FW). Statistical criteria for calibration aimed to minimize the deviation between the simulation outputs and the observed values for the variables “total above-ground biomass” (WAGT) and “grain yield” (GY). As we assumed that salinity responses are variety-specific and measurable, parameters related to salinity responses were not calibrated but given as inputs into the model. These parameters were estimated from earlier greenhouse experiments characterizing the salinity responses of the three varieties (Radanielson et al., 2018b, Table 2). The ORYZA v3 model used soil salinity data as model inputs and used daily linear interpolations to simulate daily salinity dynamics from the weekly and the fortnightly soil salinity data available. Initial soil salinity ranged from 0.9 to 1.5 dS m^−1^. As the soil salinity dynamics were supplied as inputs, no effect of soil type was considered in the variation of soil salinity.

**Table 2:** List and values of crop model parameters for scenario analyses. Parameters in combination are the parameters that change among the virtual varieties considered. The Resilience parameter (R) is the rate of decrease of photosynthesis and transpiration at the point when a 50% reduction occurs (aSalt). The Tolerance parameter (T) is the level of salinity at which the crop transpiration (bTR) and the photosynthesis (bPN) are reduced by 50%. The Phenology parameters (P) are the durations during vegetative (Vg) and reproductive (Rp) stages. Virtual combinations are the possible combinations (RxTxP) obtained with the 5 values of R, the 10 values of T and the 6 values of P. Reference varieties are characterized by values of parameters related to their responses to salinity.

Variety and combinations	Resilience (R) aSalt	Tolerance (T) bTR-bPN	Phenology Vg-Rp (P, DAS)
Virtual combinations (RxTxP)	0.11 0.21 0.31 0.41 0.51	2.17–5.08 4.17–7.08 5.17–8.08 6.17–9.08 7.17–10.08 8.17–11.08 9.17–12.08 10.17–13.08 11.17–14.08 12.17–15.08 13.17–16.08	80–30 (P1, 110) 90–30 (P2, 120) 80–40 (P3, 120) 80–20 (P4, 100) 70–20 (P5, 90) 60–30 (P6, 90) 60–40 (P7, 100)
IR64	0.21	7.83–11.17	
IR29	0.31	4.17–7.08	
BRRI dhan47	0.19	12.3–16.15	

#### 2.1.3 Crop model validation

The model was run to simulate crop growth and yield of the three varieties under salt stress. Data obtained from the different saline water treatments (1W, 2W, SW) at all sites during the two seasons were used (2013 and 2014). Measured values of soil salinity in experiments 1 to 4 were used as input data in the model to inform on salt stress. It is worth noting that because measured soil salinity values were used as direct inputs to the model, no further variation of soil salinity with soil texture and structure was accounted for. However soil osmotic potential presents variation with soil salinity, thus changing soil water available for uptake by the plant as indicated in the model description by Radanielson et al. (2018a). The ability of the model to simulate the observed crop performance in Expts. 1 to 4 was evaluated by comparing the simulated and observed values of total above ground biomass (WAGT) and rice yield (GY) following a standard protocol for model validation (Li et al., 2009).

### 2.2 Model scenario analysis

Scenario simulations were developed for Satkhira, Bangladesh (1984–2014), to explore the performance of different combinations of crop traits on the field-scale performance of rice. The objective was to quantify the effect of the variation of each relevant trait on crop yield, and thereby, to define strategies for improving salinity tolerance.

Simulations of different crop trait combinations were then performed with the modified version of the rice crop Model ORYZA v3 (Radanielson et al., 2018a). Virtual varieties were designed to represent a range of salinity tolerance and crop growth duration values. They were characterized based on potential yield of BRRI dhan47. Crop growth duration was changed using variation of the developmental rate parameters of the model, considering that the grain filling phase remained the same (DVRR) for the considered combination and the duration of sowing to PI (DVRJ) and PI to flowering (DVRI) were the ones reduced or delayed. Each virtual variety has a unique combination of crop phenology (development rate, DVR), salt tolerance (bPN, bTR) and salt resilience (aSsalt) (Table 2). DVR phenology parameters’ values were set to create short (P5 and P6 of 90 DAS), medium (P4 and P7 of 100 DAS) and long duration growth types (P1 to P3 ranging from 110 to 120 DAS). Two dates of sowing were used: the current sowing date used by farmers, around 1^st^ of January (D1) and an optimized date of sowing for boro rice for Satkhira region of 15^th^ of November (D2, Mondal et al., 2010, 2015). Irrigation was managed to maintain sufficient water. Salt stress conditions were maintained by using saline water for irrigation based on the specific treatment. The impact of climate variability was evaluated using daily historical climate data over the last 30 years as a model input, from 1984 to 2014 for Satkhira. River salinity in Satkhira has shown significant increases over recent years. The use of water from the river to irrigate the fields in earlier years was possible to maintain productivity. The pattern observed in variability of soil salinity during 2012 and 2013 was then used as average annual pattern to assure that salinity trend would have no effect over years. A total of 420 virtual varieties (Table 2) were then simulated for 30 years for the two sowing dates and the two crop growing conditions (Non-limited and salt stressed, under SW condition). Temporal and spatial variability of soil salinity among years and within the rice field was not considered in the scenarios. The objective was then to isolate the effect of the variation of the traits on rice crop growth and yield under a given scenario of soil salinity condition.

### 2.3 Data analysis

The model validation was performed using several statistical analysis tools. Linear regression was initially used to compare paired data points for measured and simulated aboveground biomass and grain yield. The slope (α), intercept (β), and coefficient of correlation (R^2^) of the linear regression were computed using the ORYZA Analysis Tools (IRRI, 2015). The model performance was also assessed using the Student’s *t*-test of means assuming unequal variance P(t), and using the normalized root of the mean squared error, RMSE_n_, which was calculated as follows:

(2) $$RMSE_{n}=\frac{\sqrt{\underset{i=1,n}{(\sum }{\left(S_{i}-O_{i}\right)}^{2})/n}}{\mu }$$

where S_i_ and O_i_ are simulated and observed values, respectively, and n is the number of pairs; μ, the overall mean of the observed values.

The index of model agreement (ID) was also used as a measure of the model’s performance, calculated as follows:

(3) $$ID\;=\;1-\left[\frac{\sum_{i=1}^{n}{\left(S_{i}-O_{i}\right)}^{2}}{\sum_{i=1}^{n}{\left(S_{i}-\mu \right)}^{2}}\right]$$

where S_i_ and O_i_ are simulated and observed values, respectively; μ is the overall mean of the observed values; n is the number of pairs.

In the scenario analyses, the simulation outputs for the trait combinations characterizing each virtual variety and date of sowing (D1 and D2) were analysed with a general linear regression model, using R software (R Development Core Team, 2008). Mean and standard deviation values over the 30 years of simulation were computed to evaluate variability among factors. Grain yield and relative yield were the variables considered. The relative yield (RY) was computed as the ratio between simulated yields under SW conditions (YS) and the simulated yield under non-limited conditions (YP). Identification of the best crop parameter combinations was based on the relative yield of each combination under saline conditions (SW).

For the best combination identified, the effect of variation in each crop model parameter related to salinity response was evaluated using the relative change compared with the simulated yield of a reference variety (IR64). This approach allowed classification of the current varieties IR29 and BRRI dhan47 and quantification of relative yield change expected with salinity tolerance improvement based on IR64 characteristics.

## 3 Results

### 3.1 Genotypic variability in crop biomass and yield under different salinity treatments

**Fig. 1:**
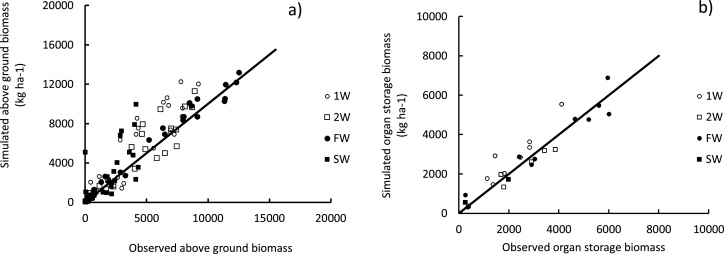
Simulated and observed total above ground (a) and organ storage (b) biomass. Each point represents measured and simulated values from irrigation water treatments in Expts. 1 to 4. The line represents the 1:1 linear relationship between observed and simulated values. FW, control treatment with irrigation with freshwater ; SW, treatment with continuously irrigation with saline water; 1 W, treatment with irrigation with alternate one week fresh and one week saline water in Expts. 1 and 2 and irrigation with a 1:1 mixture of fresh and saline water in Expts. 3 and 4 ; 2 W, treatment with irrigation for two weeks with freshwater and one week with saline water in Expts.1 and 2 and irrigation with a 2:1 mixture of fresh and saline water in Expts. 3 and 4.

The four treatments of irrigation management resulted in different levels of soil salinity for each field, with a mean value ranging between 1.1 and 7.7 dS m^−1^, respectively, for FW (Expt. 2) and SW (Expt.1, Table 1). Responses of the three varieties to these treatments were compared based on total above ground biomass estimated at flowering and physiological maturity (WAGT_fl_ and WAGT_pm_, kg ha^−1^) and on grain yield (GY, kg ha^−1^). Genotypic and treatment effects as well as their interactions were significant. WAGT_fl_ varies from 1936 (±183) to 6107 (±1130) kg ha^−1^, respectively, for IR29 under SW, and BRRI dhan47 under FW. Similarly, differences between genotypes, treatments and their interactions were significant for WAGT_pm_. The smallest and largest values were, respectively, observed for IR29 under SW (2013 kg ha^−1^) and IR64 under FW (13,130 (±600) kg ha^−1^). Grain yield (GY) was also significantly different when compared across genotypes and treatments, with variation ranging from 313.7 for IR29 under SW, to 6467 (±517) kg ha^−1^ for IR64 under FW (Fig. 1). BRRI dhan47 had the highest grain yield (838 (±383) kg ha^−1^) among varieties under severe stress (SW).

### 3.2 Model validation in simulation of genotypic variability in rice responses to salinity

Simulated WAGT and GY with the improved version of ORYZA v3 showed good agreement with the observed values for the three varieties used in this study. The overall model index of agreement was 0.94 to 0.96 corresponding to an RMSE_n_ of 34.6 and 22.8%, respectively, for WAGT and GY (Table 3). A similar level of accuracy was observed among varieties, with RMSE_n_ lower than 40%. Simulated WAGT showed higher values for RMSE_n_ ranging between 32.9% and 35.9%, corresponding to an RMSE of 1204 to 1212 kg ha^−1^. An overestimation of the simulated WAGT for IR29 and IR64 was observed as indicated by the slope of the linear relationship between the simulated and the observed values of WAGT for these varieties (1.05 and 1.01, respectively, Table 3).

**Table 3:** Validation statistics: observed versus simulated aboveground biomass (WAGT) and yield (GY) of the three rice varieties.

Sites	Varieties	Variables (kg ha^−1^)	n	P(t*)	β	α	R^2^	RMSE	RMSE_n_ %	ID
Infanta	IR29	WAGT	33	0.07	390	1.05	0.93	1459	33.6	0.84
		GY	9	0.05	582	0.96	0.91	821	24.7	0.73
	IR64	WAGT	33	0.08	293	1.01	0.93	1436	35.6	0.83
		GY	10	0.03	851	0.85	0.89	794	29.4	0.70
	BRRI dhan47	WAGT	24	0.09	588	0.93	0.92	1212	35.9	0.83
		GY	11	0.39	−88	0.92	0.97	329	15.9	0.95
Satkhira	BRRI dhan47	WAGT	22	0.02	169	0.95	0.93	1204	32.9	0.85
		GY	4	0.27	861	0.74	0.97	596	16.3	0.89
	Overall	WAGT	112	<0.001	−26	1.01	0.93	1354	34.6	0.94
		GY	34	<0.001	465	0.92	0.93	632	22.8	0.96

Note: *n*, number of data pairs; P(t^*^), significance of Student’s paired *t*-test assuming non-equal variances; α, slope of the linear regression between simulated and measured values; β, y-intercept of linear regression between simulated and measured values; R^2^, root square of linear correlation coefficient between simulated and measured values; RMSE, absolute root mean squared error; RMSE_n_, RMSE normalized by the mean of the observed measurements as percentage; ID, model index of agreement; WAGT, above-ground biomass.

The RMSE in simulated yield ranged from 596 to 821 kg ha^−1^, corresponding to an RMSE_n_ of 15.9 to 29%. This was within the range of the standard deviation of the observed yields (data not shown), indicating acceptable model performance (Gaydon et al., 2017). Considering the different salinity conditions imposed with the irrigation water treatment, the model was able to predict the difference in salinity responses between the tolerant variety BRRI dhan47 and the other two varieties IR29 (sensitive) and IR64 (intermediate). BRRI dhan47 consistently had higher yields than IR29 and IR64 under salt stress conditions. In contrast, their yields simulated under non-stressed conditions showed no significant differences.

### 3.3 Simulated yield variability among virtual combinations for phenological traits

Virtual varieties were compared based on yield under control conditions (YP), and yield under continuous irrigation with saline water (YS). A relative yield (RY: ratio of YS to YP) was computed to evaluate the effect of the severity of stress on yield losses. A significant effect of sowing date was observed on YS, YP and consequently on RY (Table 4) among the varieties representing different combinations of phenology and salinity response traits values. Interaction between sowing date and variation in phenology traits was also significant. Variation in traits related to salinity response (Tolerance (bTr and bPn) & Resilience (aSalt)) did not affect YP.

**Table 4:** Analysis of variance of simulated yield among the traits tested and dates of sowing. MSE mean square error, df degree of freedom, SW, irrigation continuously with saline water.

Factors	Df	Yield under control conditions (YP)		Yield under SW (YS)	
		MSE (10^9^)	F value	MSE (10^9^)	F value
Phenology (P)	6	19.10	55888.2***	0.435	1199.7***
Tolerance (T)	11	0.0005	1.6	0.272	749.8***
Resilience (R)	4	0.0006	2.0	0.136	374.3***
Date of sowing (D)	1	1.43	4177.8***	22.5	61968.8***
DxP	6	0.15	440.8***	0.383	1056.1***
Error	24287	0. 0003		0.0003	

*** Significant at *P* <  0.001.

**Fig. 2:**
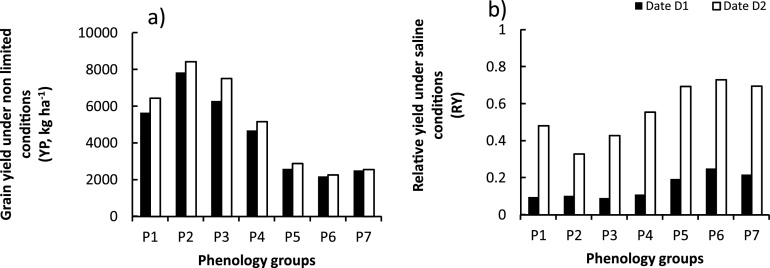
Scenario analysis: simulated grain yield under control conditions (YP) and relative yield (YR) under saline conditions among the phenology groups tested and dates of sowing. Date D1, 1^st^ December; Date D2, 15^th^ November. Each bar represents the mean value of simulated YP and calculated RY over 30 years (1984–2014) at Satkhira, Bangladesh. Phenology groups are long duration varieties (P1 to P3 of 110–120 DAS), short duration (P5 and P6), medium duration (P4 and P7).

YP showed large variability among the virtual varieties with an overall mean ranging from 2179 (±3.72) to 8420 (±11.29) kg ha^−1^, corresponding to the group of varieties defined with phenology P6 and P2, respectively (Table 2 and Fig. 2a). Crops sown earlier on 15th November (D2) consistently showed higher YP over crops sown on 1st December (D1).

The group of virtual varieties with phenology P6 (short duration of 90DAS) showed the highest relative yield (0.73 and 0.25, respectively, under D2 and D1), suggesting ability to tolerate salinity (Fig. 2). In contrast, the group of combinations with longer growth duration of 110–120 DAS (phenology P1–P3) were the most sensitive, with relative yield decreasing to 0.43 and 0.09, respectively, with D1 and D2 (Fig. 2). The combinations with phenology P3 (120DAS), however, showed the maximum overall mean YS. P1 and P3 showed more than 50% yield loss under D2 and more than 90% yield loss under D1, suggesting that long duration varieties particularly with longer reproductive stage are exposed to increasing salt stress for longer duration and hence, are more sensitive.

### 3.4 Variability of simulated yield with group of traits combinations

Under saline conditions (SW), the group with the highest relative yield, which showed the lowest yield loss, was not always the highest yielding varieties. Simulated YS ranged from 2.03 (±0.38) kg ha^−1^ to 4283 (±236) kg ha^-1^, corresponding respectively, to the combination (P3 x T1 x R5) sown on 1^st^ January and (P3 x T12 x R5) sown on 15^th^ November. The combination (P3 x T1 x R5) was the most sensitive combination with a RY of 0.003 (±0.00) for 1st December sowing date. The combination (P3 x T12 x R5) presented low to medium level of tolerance with a RY of 0.18 (±0.009) to 0.57 (±0.03), respectively, for sowing date of 1st January and 15th November. The most tolerant combination was (P6 x T12 x R5) with RY of 0.81 (±0.02) when it was sown on 15th November.

**Fig. 3:**
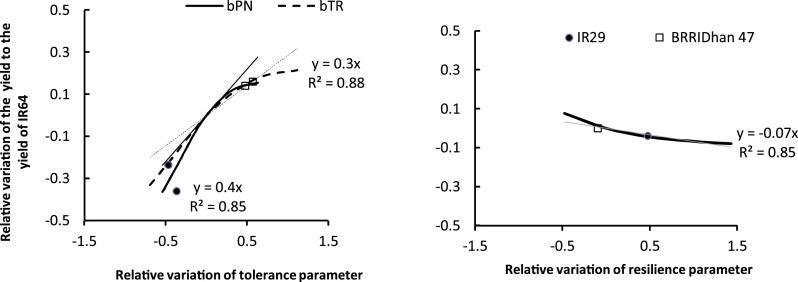
Scenario analysis: Changes in relative yield with variation in salinity parameters of tolerance (bTR and bPN) and of resilience (aSalt). The circle and the square symbols represent the relative values of salinity parameters at the relative yield values expected for IR29 and BRRI dhan47, respectively. The lines represent the linear relationship between the change in relative yield (y-axis) and the variation in aSalt (grey line), bPN (dashed line) or bTR (black line) (x-axis). The change in relative yield is compared with the mean relative yield of the reference variety IR64 under the SW treatment. The relative change in the considered traits (increasing from 0 to +1.5 or decreasing from 0 to −1.5) is relative to the reference represented by IR64 parameter values.

Considering the effect of salinity response traits on YS and RY variability, significant effects were observed with a higher contribution from the tolerance traits than the resilience traits (Table 4). Within the group of combinations of salt tolerance traits, an increase of yield was obtained with the increase of tolerance (Fig. 3). Among combinations with higher tolerance, the effect of the variation in resilience was not significant. However among combinations with medium or lower tolerance, an increase in yield was observed with decrease of the resilience trait values (Fig. 3).

To quantify the effect of individual variation of salinity tolerance traits on RY, a linear regression analysis was performed between the relative change in yield and the relative change of the trait compared to a reference variety (IR64). As indicated in Fig. 3, two phases of responses were observed. The first phase was a linear relationship up to a value of the tolerance at which no further responses in yield were obtained. This corresponded to variation of the trait from the current value of the variety IR64 (defined as 7.83 and 11.08 dS m^−1^, Table 2) indicated at the origin of the x axis, to a value of 1 which is an increase by 100% of the current tolerance level (∼15–22 dS m^−1^). The second phase was revealed as a plateau corresponding to an optimum value of tolerance, presenting no increase in yield gain beyond this level.

This linear framework confirms the characteristics of the sensitive variety IR29. This variety showed yield loss compared to the reference IR64, suggesting it has lower tolerance than IR64. The current characteristics of BRRI dhan47 were optimum for the tolerance trait, close to the plateau phase. BRRI dhan47 and IR64 showed similar resilience. As most breeding strategies aim to use yield as a target trait to evaluate improvement, the approach we have presented attempts to introduce a trait-based philosophy in considering salinity tolerance and in focussing on processes such as transpiration and photosynthesis which are physiological determinants of biomass production and thus yield formation.

This simulation output analyses illustrated that a relative increase of 1% over the current tolerance of IR64 would result in 0.3–0.4% yield gain (R^2^ = 0.85–0.88, p < 0.001). Lower change was observed in the variation of the resilience trait values. About 0.07% yield gain was obtained with an improvement of the resilience trait (associated with slower rate of decrease) by reducing its value by 1% (R^2^ = 0.85, p < 0.001). Compared with the tolerant variety BRRI dhan47 and under the environmental conditions of this simulation, an improvement of resilience could help in breeding a new generation of salt-tolerant rice varieties. This improved tolerance would have benefits under conditions with higher salinity than was considered in the present simulations. To conclude then, the contribution of the improvement of the tolerance to salinity of photosynthesis and transpiration by 1 dS m^−1^ would result to an increase of 337–400 kg of yield asuming a btr value of reference of 7.83 dS m^−1^.

## 4 Discussion

### 4.1 ORYZA v3 model representation of salinity tolerance in rice

The rice crop model ORYZA v3, with its new functions accounting for salt stress effects on rice growth and yield, performed well in simulating total aboveground biomass (WAGT) and grain yield (GY) with a RMSE between observed and simulated values of 1354 and 632 kg ha^−1^, respectively (Table 3). These values of RMSE are within the standard deviation of the field measurements for WAGT and GY used to calibrate and validate the model. Similar ranges of RMSE have been reported for the model in simulating different fertilizer and irrigation management effects on rice yield (e.g. Bouman and van Laar, 2006; Belder et al., 2007; Boling et al., 2011; Sudhir-Yadav et al., 2011, 2012). The new functions of salinity in the rice crop model ORYZA v3 have expanded its domain of application (Radanielson et al., 2018a). The model was able to represent the variability among three contrasting rice varieties with a consistent ranking of their performance under four gradients of saline conditions ranging from non-stress to severe stress up to 14 dS m^−1^ (FW, SW, 1W, 2W; Table 1, Fig. 1). This implies that genotypic parameters used to characterize the three varieties in the model represented the observed variability of the responses to salinity expressed by the three varieties under the conditions of the sites of study. These parameters were genotype-specific, able to reproduce the response of each variety to the variability of its environment. The representation of salinity effect may not be exhaustive over all processes affected by the stress, such as phenology, the increase of respiration, the change in biomass partitioning, and changes in yield components (Lutts et al., 1995; Asch et al., 1999; Karlberg et al., 2006). Decomposing the effect to several factors affecting each of these components would improve the model accuracy and allow a detailed understanding of the salinity effect at all scales. For instance by calibrating the crop parameters of the model related to drought response, as the FTSW and the threshold of soil water tension affecting leaf growth (Li et al., 2017; Wopereis et al., 1996), the accuracy of the model has been improved by capturing 10% more of the variability of the observed values (Supplementary Fig. 2). This suggests then that characterizing each of the varieties responses to drought would contribute to improved salinity response representation of these varieties. We recognized as well the limitation of the model structure and representation that would limit reporting of the result as absolute values in addition to the uncertainties around the soil salinity measurements and the crop growth measurements to estimate these parameters. These have led to variation in the accuracy of the model simulations among the salinity levels. The modelling approach within the modified version of ORYZA presented a sufficiently simplistic description which allowed the integration of the stress effect with the whole plant function and with yield and biomass production, the main focus of the present work. A reconstruction of the model would be needed for instance, if the model were to be used to link the salinity tolerance, which is accomplished by different traits, to each trait genetic background as explored for instance for the stay green trait of maize and sorghum (Borrel et al., 2014; Kholov et al., 2014)

### 4.2 Factors driving yield variability under saline conditions

#### 4.2.1 Management and environmental factors

Simulated yield under non-stress (YP) and saline conditions (YS) revealed large variability, with the effect of sowing date contributing significantly to this variability (Table 4). Mean square error (MSE) among the sowing dates explained more than 90% of the total mean square error of YP and YS. Early sowing (15th November) can result in higher yield and lower yield losses due to salinity (Fig. 2). In contrast, late sowing (1st December) results in lower grain yield and an exposure to higher salt stress during the cropping season. Early sown crops benefit from higher radiation around maximum tillering stage (end December to early January) and escape the rise in salinity later in the season (around 15th–30th April; Sarker et al., 2012). This confirmed that timing of planting is one of the important strategies for adaptation to salinity in coastal areas. Temporal shifts in the cropping calendar are among the best adaptation strategies to deal with the increasing occurrence and severity of abiotic stresses due to climate change in rice systems (Matthews et al., 1997; Li et al., 2015; Gaydon et al., 2018). For the region of Satkhira, the 15^th^ November has been defined as the optimum date for sowing dry season rice (Mondal et al., 2010, 2015; Gaydon et al., 2018). It is, however, important to note that early crop establishment must consider the crop rotation and potential harm from cold stress during the dry season (Saha et al., 2015). A parallel ongoing work employed modelling to explore crop management opportunities considering these complex interactions between environmental factors and the extent of variety tolerance in order to increase cropping systems’ productivity in the coastal zones of Bangladesh (Gaydon et al., 2018).

#### 4.2.2 Phenology traits

Phenology traits were the second source of variability for the simulated yield under non-stress conditions (YP). YP correlated positively with intercepted solar radiation. Long crop duration, with a longer phase of maximum radiation interception was expected to result in higher yield potential, assuming no negative effects of high temperature stress during crop growth and reproductive development (Casanova et al., 2002; Yang et al., 2008). Under saline conditions, the interaction between sowing date and crop phenology was the second most important source of variability for YS. Differences in date of sowing resulted in differences in soil salinity dynamics during the crop growth. The crop sown on 1st December experienced stress during most of the sensitive stages: panicle initiation and flowering, but sowing on 15th November would only expose the crop to salinity during the grain filling stage, which is relatively more tolerant of salt stress in rice (Moradi et al., 2003). Salinity had higher impacts on rice yield during panicle initiation and flowering time than after flowering (Heenan et al., 1988; Lutts et al., 1995; Khatun et al., 1995). River salinity in Satkhira increased progressively during the season, starting from February until desalinization phase at the beginning of the succeeding rainy season (May-June). Similar trends were also observed in soil salinity, which surpassed 12 dS m^−1^ by end of March and increased further until the end of the dry season. The group of cultivar combinations examined with short growth duration (P6) escaped this period of higher stress when they were sown early (15^th^ November, D2) and experienced moderate stress when sown later (1st December, D1). Short duration varieties could improve productivity of rice-based systems (Matthews et al., 1997; Balwinder-Singh et al., 2015). The choice of such varieties however, carries with it the limitation of lower potential yield; even though with reduced risk of late season salt stress. Improving grain yield of these short-maturing varieties through e.g. greater biomass production and/or higher harvest index should be considered in breeding programs targeting these saline coastal zones.

#### 4.2.3 Salinity response traits

The effect of the variation of salinity traits on yield was quantified using a regression between relative changes in yield with relative changes in varietal salt tolerance. This quantitative approach is useful for conceptualizing the ability of the three contrasting varieties used in this study to perform well in the Satkhira environment. The Satkhira site is defined as a ‘slight to moderate’ salt affected area, with salinity ranging from 8 to 12 dS m^−1^ (Mondal et al., 2015). Relative to IR64, the reference variety, the sensitive variety IR29 possessed salinity tolerance traits that would result in a yield reduction of more than 30% of that obtained with IR64 under severe salinity (SW). In contrast, the tolerant variety BRRI dhan47 possesses tolerance traits that would increase yield by more than 15% over that of IR64. Under extreme conditions with soil EC > 12 dS m^−1^, IR64 has was reported to have complete yield loss as its critical threshold for 50% yield loss is around 6–7 dS m^−1^ (Moradi et al., 2003; Castillo et al., 2007). With salt stress of 6–7 dS m^−1^, IR64 would maintain its yield at a maximum of around 65% of its yield under non- stressed conditions, while BRRI dhan47 would maintain its yield at least at 85%. The salinity tolerance of BRRI dhan47 was based on the plateau phase of the relationship between trait change and yield change. This result implies that with the current saline conditions at Satkhira, the level of tolerance of BRRI dhan47 is sufficient and can improve grain yield under the current salt stress conditions compared with high yielding varieties such as IR64 or even BRRI dhan28, the highest yielding variety used by most farmers in Satkhira region in areas where salt stress is low. Assuming a linear relationship, the framework facilitates estimation of gain in grain yield per unit of improvement in the salt tolerance traits. This type of quantification could add significant value to breeding programs as it can be used to guide crop improvement and to estimate returns on investments in breeding programs in term of yield gain considering the conditions of the areas targeted for adoption. For instance, the development of BRRI dhan47 from a current high yielding variety similar to IR64 has cost around USD 50,000 (Islam M. D., Pers. comm). This cost could then be justified for improving the tolerance of IR64 by 0.5 of its current level. Assuming an adoption of BRRI dhan47 in more than 500,000 ha over 5 years, this investment would have already generated a positive benefit of 100 times in yield gain (assuming that 15% of the production gain is due to improvement in salt tolerance). Considering the predicted increase in salinity with climate change by 2050, half of this investment would be required to further improve BRRI dhan47 and similar varieties, to be suitable for future conditions. Further development of this framework would be of interest to estimate varietal improvement options with climate change, as the negative impacts of biotic and abiotic stresses are expected to worsen (Wassmann et al., 2009b). Salinity is a complex stress with complex interactions with other abiotic stresses; in addition to its spatial and temporal variability that our model scenarios have not fully considered. Salt build up in soil solution presents variation between saline soil and normal soil particularly with the use of saline irrigation water. By using soil salinity as direct inputs in the model, we did not attempt to explore variability of the environment, thus limiting the interpretation of the results to the current conditions of the study areas. The ORYZA model does not need initialization information accounting for the difference between saline and non-saline soil as soil solution salinity dynamic during crop growth is an input to the model (Radanielson et al., 2018a). In contrast with the APSIM-ORYZA model, the users would have opportunity to simulate the difference in soil salinity build up between saline soil and non-saline soil. The approach in using soil solution salinity as model inputs has simplified the complexity of the effect of salinity traits in determining yield and could be extended considering interactions with other yield limiting traits such as leaf growth and biomass partitioning.

## 5 Conclusions

Options for rice varietal improvement for salt affected areas have been identified in this study through the conjunctive use of field experimentation and the rice crop model ORYZA v3. The model parameters related to salinity tolerance and resilience predicted specific genotypic responses to salinity in rice with acceptable accuracy. Scenario analyses with the validated model suggest that early sowing with short duration varieties in salt affected areas would maintain grain yield at about 85% of its value under non-saline conditions in the Satkhira region of south Bangladesh. A novel quantitative framework was developed to evaluate the responses of yield to changes in the crop model parameters related to salinity response (aSalt, bPN, bTR), with the conclusion that a level of tolerance higher than 12 dS m^−1^ as observed in BRRI dhan47, is suitable for the current conditions in Satkhira. An improvement in the resilience of this variety would be of interest for changing patterns of salinity, driven by irrigation management strategies using fresh water. An integrative approach using the quantitative framework developed in this study, combined with studies on the genetics of salinity tolerance, and with proper characterization of target environments, would help increase the efficiency of breeding programs to develop future rice varieties with greater tolerance of salt stress. The development of such new varieties, in association with proper cropping systems management, will provide effective means to cope with climate change and sustain future rice production in less favorable areas.
