# Supplementary material for: Varietal improvement options for higher rice productivity in salt affected areas using crop modelling
Source: Field Crops Res. 2018 Dec 1;229:27–36. doi: 10.1016/j.fcr.2018.08.020 (PMC6472128; doi:10.1016/j.fcr.2018.08.020)
Supplement: Supplementary file 1 [file mmc1.docx]

Supplemental Figure 1. Salinity dynamics in the field under SW treatment irrigated continuously with water from the canal and the river from Expt 1 and 2 (a) and from Expt 2 and 3 (b).

a)

Supplemental Table 1. Simulated and observed phenology from Expt.1 and 2. Observed data are from monitoring of 6 to 12 hills per subplot in each replication. PI – panicle initiation; FL – flowering; PM – physiological maturity.

|  | Variety 1 | | | Variety 2 | | | Variety 3 | | |
| --- | --- | --- | --- | --- | --- | --- | --- | --- | --- |
| **Treatment** | **PI**  **( Obs- Sim)** | **FL**  **( Obs- Sim )** | **PM**  **( Obs- Sim )** | **PI**  **( Obs- Sim)** | **FL**  **( Obs- Sim )** | **PM**  **( Obs- Sim )** | **PI**  **( Obs- Sim)** | **FL**  **( Obs- Sim )** | **PM**  **( Obs- Sim )** |
| Control FW | 64-64 | 89-83 | 114-114 | 64-64 | 89-86 | 114-108 | 64-69 | 89-88 | 114-115 |
| Treatment 1 1W | 67-64 | 89-83 | 118-114 | 66-64 | 89-85 | 117-110 | 67-70 | 89-88 | 115-115 |
| Treatment 1 2W | 67-64 | 93-85 | 119-116 | 75-64 | 92-87 | 120-109 | 78-69 | 90-89 | 116-116 |
| Treatment 3 SW | 84-64 | 93-90 | 124-118 | 78-64 | 92-91 | 120-110 | 69-70 | 90-93 | 120-113 |

Supplemental figure 2- Simulated and observed phenology from Expt.1 and 2. Observed data are averaged values from monitoring of 6 to 12 hills per subplot in each replication per variety and per treatment

.

Supplemental Table 2. Statistical parameters of the comparison between observed and simulated mass of aboveground biomass (WAGT) and storage organs (WSO) of the three rice varieties IR64, IR29 and BRRIDhan 47 combined. Observed and simulated total above ground biomass and storage organs considered were from Expt 1 to 4. FW, control treatment, irrigated with fresh water; 1W, treatment 1 irrigated with alternate weekly fresh water and saline water, 2W, treatment 2 with alternate 2 weeks fresh water and saline water and SW, treatment 3 irrigated continuously with saline water.

| **Treatment** | Variables  (kg ha^-1^) | n | β | α | R^2^ | RMSE |  | RMSE_n_  % |
| --- | --- | --- | --- | --- | --- | --- | --- | --- |
| FW | WAGT | 30 | 524 | 0.94 | 0.96 | 1301 |  | 24.4 |
|  | WSO | 13 | -49 | 1.00 | 0.96 | 499 |  | 13.7 |
| SW | WAGT | 19 | 425 | 074 | 0.89 | 753 |  | 42.7 |
|  | WSO | 11 | 497 | 1.9 | 0.55 | 283 |  | 23.9 |
| 1W | WAGT | 31 | 1083 | 0.68 | 0.84 | 2004 |  | 51.0 |
|  | WSO | 11 | 84 | 0.71 | 0.93 | 972 |  | 42.3 |
| 2W | WAGT | 32 | 520 | 0.86 | 0.97 | 778 |  | 20.3 |
|  | WSO | 9 | -122 | 1.13 | 0.97 | 365 |  | 16.5 |
| Overall | WAGT | 112 | -25.8 | 1.01 | 0.92 | 1354 |  | 34.65 |
|  | WSO | 43 | 106.1 | 0.94 | 0.87 | 903 |  | 36.9 |

Supplemental Figure 4. Observed and simulated total above ground biomass (a) and organ storage (b) with arbitrary calibration of crop drought parameters responses. Data points represent averaged values for each variety from 3 replications under each treatment : FW, control treatment, irrigated with fresh water; 1W, treatment 1 irrigated with alternate weekly fresh water and saline water, 2W, treatment 2 with alternate 2 weeks fresh water and saline water and SW, treatment 3 irrigated continuously with saline water.
